# *Nypa fruticans* Frond Waste for Pure Cellulose Utilizing Sulphur-Free and Totally Chlorine-Free Processes

**DOI:** 10.3390/molecules27175662

**Published:** 2022-09-02

**Authors:** Evelyn, Sunarno, David Andrio, Azka Aman, Hiroshi Ohi

**Affiliations:** 1 Department of Chemical Engineering, University of Riau, Pekanbaru 28293, Indonesia; 2 Kerinci Mill, PT. Riau Andalan Pulp and Paper, Pangkalan, Kerinci 28300, Indonesia; 3 Faculty of Life and Environmental Science, University of Tsukuba, 1-1-1 Tennodai, Tsukuba 305-8572, Japan

**Keywords:** *Nypa fruticans* fronds, cellulose, soda cooking, peroxymonosulfuric acid, alkaline hydrogen peroxide

## Abstract

The search for alternative methods for the production of new materials or fuel from renewable and sustainable biomass feedstocks has gained increasing attention. In this study, *Nypa fruticans* (nipa palm) fronds from agricultural residues were evaluated to produce pure cellulose by combining prehydrolysis for 1–3 h at 150 °C, sulfur-free soda cooking for 1–1.5 h at 160 °C with 13–25% active alkali (AA), 0.1% soluble anthraquinone (SAQ) catalyst, and three-stage totally chlorine-free (TCF) bleaching, namely oxygen, peroxymonosulfuric acid, and alkaline hydrogen peroxide stages. The optimal conditions were 3 h prehydrolysis and 1.5 h cooking with 20% AA. Soda cooking with SAQ was better than the kraft and soda process without SAQ. The method decreased the kappa number as a residual lignin content index of pulp from 13.4 to 9.9–10.2 and improved the yields by approximately 6%. The TCF bleaching application produced pure cellulose with a brightness of 92.2% ISO, 94.8% α-cellulose, viscosity of 7.9 cP, and 0.2% ash content. These findings show that nipa palm fronds can be used to produce pure cellulose, serving as a dissolving pulp grade for viscose rayon and cellulose derivatives.

## 1. Introduction

*Nypa fruticans* (nipa palm) is a widely grown palm on peat soils and wetlands in several countries, including India, Myanmar, Thailand, Malaysia, Indonesia, Borneo, Philippines, the Ryukyu Islands, Papua New Guinea, countries in West Africa, the Solomon Islands, and northern Australia [1,2]. Previous studies also revealed that it has several advantages, such as the use of its leaves as traditional thatching materials [3]. Furthermore, this plant has been utilized for food crops in agriculture, such as the sap, which is often used for the production of toddy, vinegar, and sugar. It has also been used to produce fruit preserves, which serve as a local dessert due to their high sucrose, glucose, and fructose content [4,5]. The sap is considered a potential feedstock for ethanol or butanol production [3,5]. It can serve as a raw material for the production of medium-density fiberboards [6], heavy metal adsorbents [7], pulping materials [8], cellulose derivatives [9], and fuels [3]. The utilization of nipa palm for medicine has also been registered [10]. However, most of its parts are left as residues and can serve as a biomass source for fuels or producing new materials. It has also been considered a source of biomass for renewable energy, along with oil palm [11]. A previous study revealed that its total cultivation area in Indonesia is approximately 700,000 ha [12]. 

At present, non-wood plant fibers are often considered an important source of materials for the production of pulp and its derivatives [13]. They can be classified into agricultural residues including sugarcane bagasse, corn stalks, rice straw, wheat straw, and cereal straw, as well as natural growing plants, such as bamboo, reeds, and sabai grass. These fibers can also be categorized as non-wood crops, such as jute, cotton fiber, ramie, and cotton linters [14]. A previous study revealed that 80% of China and India’s total pulp was obtained from non-wood materials [15]. The utilization of their fibers was facilitated by the rapid growth of the pulp and paper industry, a shortage of wood fibers, the level of technical equipment, environmental contamination, and their abundant availability [14,15]. Therefore, it is important to find suitable resources, the most efficient methods, and the most environmentally friendly production techniques. 

**Figure 1:**
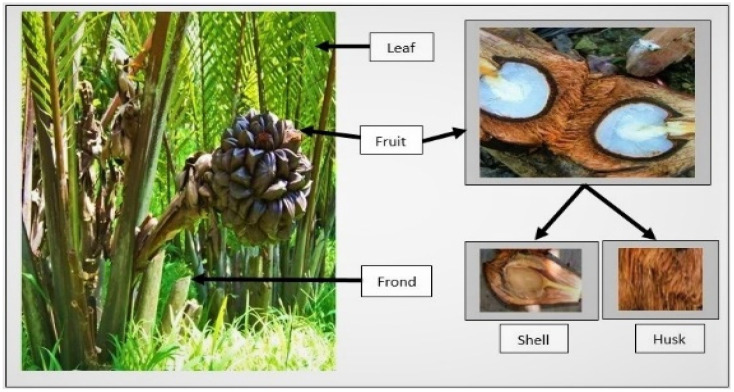
Potential parts of the nipa palm as raw materials for fuels and chemicals.

Lignocellulosic raw materials containing ≥34% α-cellulose are considered suitable for pulp and paper production [16]. Previous studies reported that different parts of nipa palm including the fronds, husk, shell, and leaf have an α-cellulose content of 28.9–48.2%; hence, they are potential raw materials for fuels and chemicals [5,17], as shown in Figure 1. The slenderness ratio, namely the fiber length/fiber diameter of its fronds and petiole, is very high compared to other non-wood fibers, such as bagasse and jute fiber, which indicates that it has high strength properties [1,18]. 

Dissolving/bright pulp is currently an important commodity in the global market, which contains >90% pure cellulose, <6–7% hemicellulose, and a low lignin content with a high brightness of >90% [19,20]. It is often used in the production of textile fibers, such as viscose rayon and lyocell as well as other derivatives, including cellulose nitrate, cellulose acetate, carboxymethylcellulose, and ethylcellulose. The high cellulose content can be obtained through the addition of a prehydrolysis step to extract most of the hemicelluloses before kraft, alkaline sulfite, or soda cooking. The process is then continued with bleaching or purification. The separated hemicellulose, namely hexose and pentose, can potentially be converted into valuable products, such as furfural, xylitol, and ethanol, which are often called potential biorefinery units [21].

Several studies explored the beneficial effect of anthraquinone (AQ) as a catalyst in the pulp and paper industries [22,23]. Furthermore, the mechanism of AQ catalysis is illustrated as a redox cycle. AQ is reduced to anthrahydroquinone (AHQ) by carbohydrates in the wood, and the product is oxidized back to the anthraquinone through a reaction, which involves reducing the active site (ß-*O*-4) in lignin during alkaline cooking [24]. Several studies also reported the benefits of AQ addition in pulping, namely a lower kappa number and improved pulp yield for kraft/soda cooking of hardwood and non-wood materials [25,26,27,28]. Utami et al. [29] revealed the advantages of prehydrolysis followed by soda with soluble anthraquinone (soda-SAQ) cooking over kraft cooking for *Acacia crassicarpa*, namely a higher pulp yield of 1.8% and a higher brightness level by 3.8–3.9 points. Jahan et al. [30] also stated that the method led to additional decreases of 1.2–6.3 points in the kappa numbers of *Acacia auriculiformis* compared to the kraft and soda processes after bleaching. These studies reported higher yields of soda-SAQ compared to kraft cooking, namely by 2.8–4.3%. However, Salaghi et al. [31] did not observe the advantages of prehydroyzed soda-SAQ over prehydrolyzed kraft for *Eucalyptus globulus* wood. All of these dissolving pulp studies only used hardwood materials, and hence no study has compared kraft, soda, and soda-SAQ pulping to produce purified pulp or dissolving pulp using non-wood materials. 

There are only a few studies on the potential of totally chlorine-free (TCF) bleaching with peroxymonosulfuric acid (P_sa_) to produce high-quality dissolving pulp [23,24,25]. Soda-SAQ cooking and TCF bleaching are considered environmentally friendly due to the sulfur-free process in the cooking and reduction in the halogenated organic compounds emissions during bleaching [32,33,34]. Jahan et al. [8] and Dewi et al. [12] evaluated *Nypa fruticans* wastes, namely nipa palm fronds (NPFs) and petioles, as pulping materials without prehydrolysis and bleaching. A previous study obtained a low pulp yield of 36.2–37.2% based on the raw materials as well as a high residual lignin content of 27.2–29.1 using the soda-AQ and kraft processes because the conditions were not optimal. Other studies only performed soda processing on nipa palm petioles without the prehydrolysis and bleaching processes, and the results showed a 27.9% yield and 7.2 pulp lignin content. This implies that there are no reports on the production of pure cellulose as a dissolving pulp grade from nipa palm using a process which includes the prehydrolysis and purification stages. The final properties and performance of pure cellulose/derivatives were affected by the raw material, processes, and other previously reported factors. Therefore, this study aims to find efficient and safe methods to produce dissolving pulp from NPF fibers.

NPFs obtained as agricultural residues were processed using prehydrolysis followed by sulfur-free cooking and TCF bleaching to produce pure cellulose as dissolving pulp grades. The chemical characterization of the sample was carried out along with the determination of the optimum prehydrolysis conditions. The possibility of pure cellulose production using a soda process with an AQ-catalyst (SAQ) and modified P_sa_-TCF bleaching was also performed. This is the first study to report the production of pure cellulose from NPF fiber using these processes. Therefore, this study aims to: (i) determine and compare the chemical composition of NPF used for pure cellulose; (ii) investigate the effect of prehydrolysis time of 1–3 h at 150 °C on kappa number, which comprises the residual lignin content and pulp yield after soda-SAQ cooking; (iii) compare the effect of cooking methods, namely kraft, soda, and soda-SAQ at 160 °C, 1–1.5 h cooking time, and 13–25% active alkali/AA dosage on pulp yields and kappa number; and (iv) apply three stages of modified P_sa_-TCF bleaching, namely oxygen, P_sa_, and alkaline hydrogen peroxide to the selected soda-SAQ pulp and compare the product to the dissolving pulp standard.

## 2. Results and Discussion

### 2.1. Potential Characteristics of Nypa fruticans Fronds for Pure Cellulose

Table 1 shows that NPFs contain 61.3 ± 2.4% holocellulose, 24.0 ± 1.9% pentosan, 17.5 ± 0.7% acid-insoluble lignin, 0.8 ± 0.5% acid-soluble lignin, 1.5 ± 0.6% extractives, and 16.5 ± 2.2% ash. Furthermore, a previous study on the characterization of NPFs for pulp showed similar results for hollocellulose, pentosan, lignin, and extractives [17]. High carbohydrate contents of lignocellulosic materials have the potential to be exploited as raw materials for pulp production, fuels, and chemicals. The obtained 60% holocellulose content is comparable to that of several non-wood plants, such as vine stem [35], grapevine stalks [36], rice, wheat straw [37,38,39], vine shoots [40], tobacco, cotton stalks [41], barley straw [41], and sugarcane whole bagasse [42]. 

**Figure 2:**
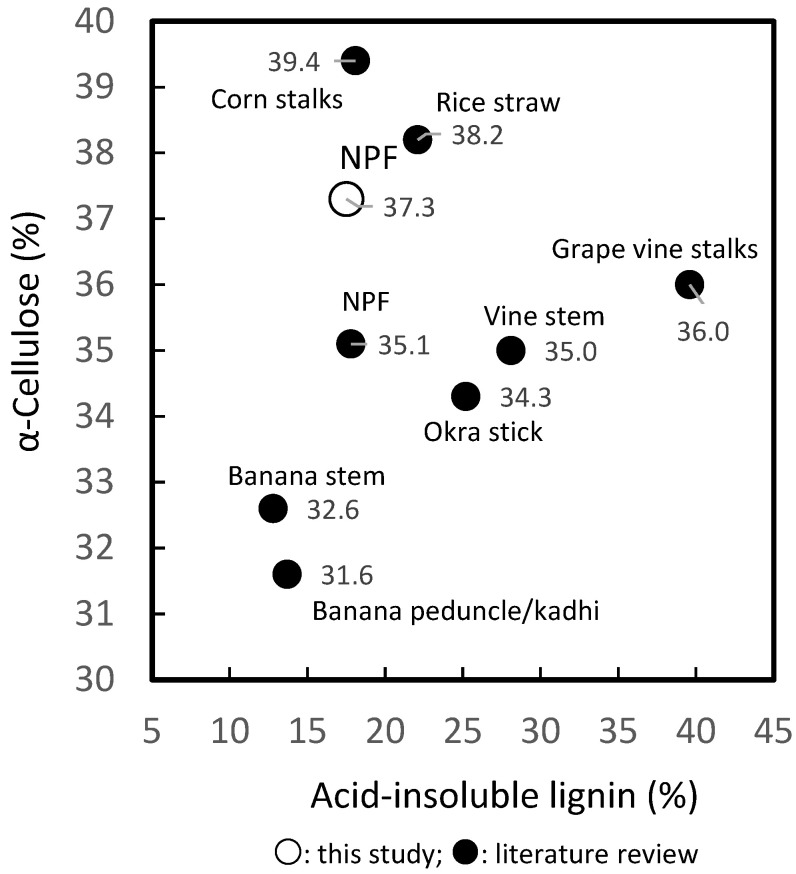
Comparison of α-cellulose and acid-insoluble lignin contents of *Nypa fruticans* fronds with those of other non-wood plants reported in the literature.

The α-cellulose content of NPF in this study was 37.3 ± 2.1%, which is also comparable to other non-wood species. Figure 2 shows that a range of 31.6–38.2% was recorded for banana peduncle/kadhi and stem [43], okra sticks [44], and rice straw [14,45]. Furthermore, the α-cellulose content of bamboo [46], oil palm fronds [47], date palm rachis [48], softwoods [49], and most hardwoods [50] is 40–56%, which is higher than non-wood materials. 

The acid-insoluble lignin content of NPF is similar to corn stalks [51], oil palm fronds (15.2%) [47], rice straw and banana stem/peduncle [43,45], but lower compared to vine stem [35], grapevine stalks [36], okra stick [44], and dhaincha (23.2%) [52], as shown in Figure 2. Tamunaidu and Saka [17] revealed that various parts of nipa palm contain a syringyl (S)-, guaiacyl (G)-, or *p*-hydroxyphenyl (P)-type lignin, and a large S to G ratio. The proportion of S units is greater than that of G units of lignin, which indicates that the wood material can easily be delignified [49]. These studies also showed that the chemical composition of all parts of nipa palm was very similar to that of oil palm. The acid-soluble lignin content obtained in this study using the TAPPI method at 205 nm and 110 L/g cm absorption coefficient is lower than the values recorded in previous studies for the same material, namely 0.8 ± 0.5% vs. 1.9% [17]. The differences in the measured values using the UV-spectrophotometric method are caused by the biomass feedstock, solubility in the solvent, differences in absorbance of the G-S-P type of lignin, and experimental factors [53]. However, the acid-insoluble lignin content is higher than the acid-soluble content in the NPFs, which contributed to the total lignin. Future studies can include a determination of the absorption coefficient, because it is important for biorefinery.

Table 1 shows that the ash content of NPF is higher than that of other non-wood plants, but it is relatively comparable to banana plant stem [54] and rice straw, namely 18.3% and 17.2%, respectively [37]. Its presence in silica can cause a variety of operational problems in dissolving pulp processes as well as limiting its uses, and hence it must be removed [16]. The silica content was not determined in this study, but previous investigations showed that it is comparatively low compared to the total ash content [17]. The extractives are also relatively low and comparable to Jahan et al., namely 1.7% [17]. 

In conclusion, NPF can potentially be used for the production of cellulose derivatives as well as biofuels and chemicals due to its α-cellulose and lignin content. The high α-cellulose content is expected to produce pulp with good mechanical properties, while relatively low lignin content often requires short reaction times or small amounts of reagent [39]. Therefore, this study examined the biomass’s suitability for the production of pure cellulose as a dissolving pulp. 

### 2.2. Effect of Prehydrolysis Time on Residual Lignin Content and Pulp Yield after Cooking

**Figure 3:**
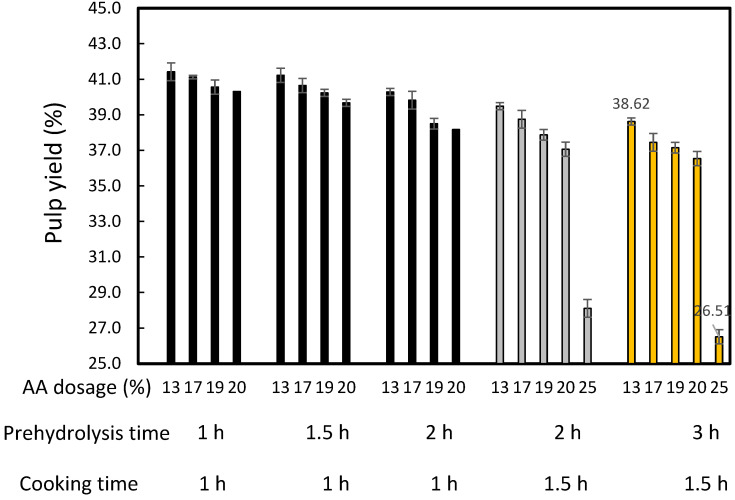
Effect of prehydrolysis time on the pulp yield of *Nypa fruticans* frond soda-SAQ pulp. The values presented are averages ± standard deviations.

**Figure 4:**
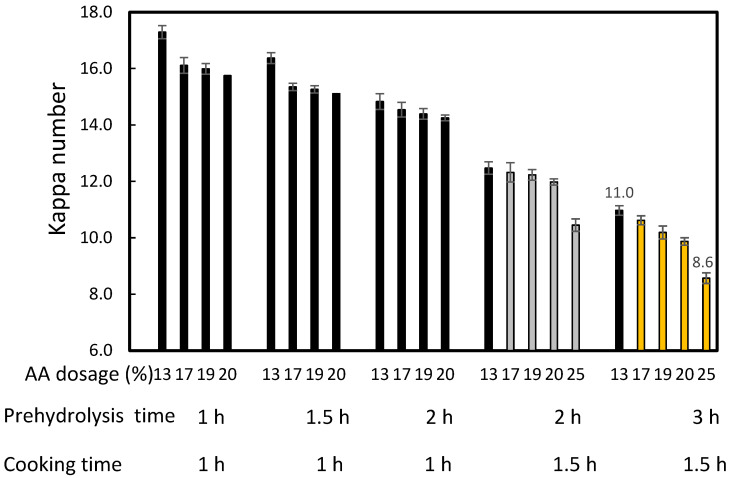
Effect of prehydrolysis time on the kappa number of *Nypa fruticans* frond soda-SAQ pulp. The values presented are averages ± standard deviations.

Figure 3 and Figure 4 show that increasing the time of prehydrolysis from 1 h to 2 h when cooking for 1 h contributed to a decrease in kappa number and pulp yield. These conditions also caused an increase in the active alkali (AA) dosage from 13% to 20%. Similar results were obtained after increasing the prehydrolysis time from 2 h to 3 h and the AA dosages when cooking for 1.5 h. The combination of prehydrolysis and soda cooking causes the hemicellulose dissolution of non-wood lignocellulosic materials and delignification. Extension of the reaction time also contributed to the decrease in kappa number.

There are no studies on the production of dissolving pulp using NPF as the raw material. Jahan et al. [6] studied soda-AQ pulp production with a 6 L/kg liquor to solid ratio, 18% AA, and 170 °C maximum cooking temperature. The findings showed that a yield of 36.2–37.2% was obtained with a kappa number of 27.2 and 29.1. However, these results cannot be compared because the processes were carried out without the prehydrolysis stage. The findings of Dewi et al. [12] with nipa palm petioles cannot also be compared. This implies that the effect of prehydrolysis time on the NPF pulp yield and kappa number after cooking was first reported in this study. No attempts were carried out to measure the pulp yield and kappa number after the prehydrolysis stage; nevertheless, Figure 3 shows quite good cellulose yields at long prehydrolysis times with low alkali dosages. Additionally, Figure 4 illustrates the positive effect on kappa number of increasing the prehydrolysis time. However, a decrease in these parameters has previously been reported with 1.0–7.0 h prehydrolysis time using Moso bamboo and oil palm empty fruit bunches [30,34,55]. The low pulp yield from the process was caused by the removal of hemicellulose [55]. Harsono et al. revealed that extension of the time did not affect the complete delignification and dissolution of hemicellulose [34]. The effect of AA on decreasing the kappa number and pulp yield in various AA dosages was also observed in the previous studies [30,32,34], which indicates that the increase in AA led to lignin removal. 

Prehydrolysis for 3 h and cooking for 1.5 h were better for lignin removal than the other conditions, namely 1–2 h prehydrolysis and 1–1.5 h cooking. 

### 2.3. Effect of Cooking Methods on Pulp Yields and Kappa Number

**Figure 5:**
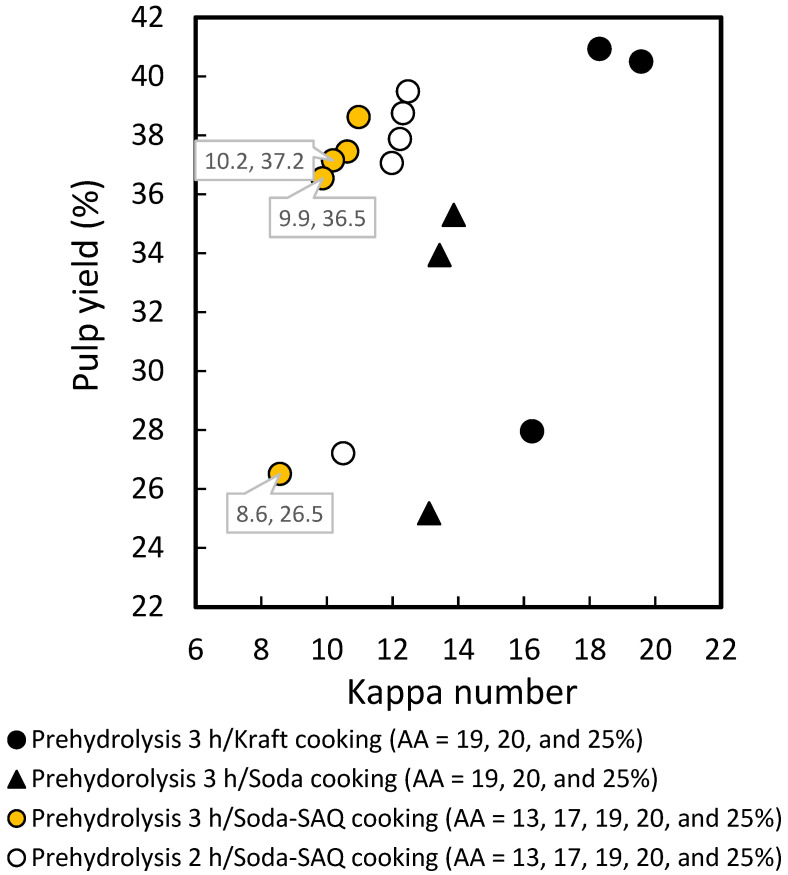
Comparison of kappa number and pulp yield in prehydrolysis cooking of *Nypa fruticans* fronds between kraft, soda, and soda-SAQ processes.

A comparison of the prehydrolysis soda-SAQ cooking with the prehydrolysis kraft and soda cooking methods is presented in Figure 5. Soda-SAQ cooking (●) reduced the kappa number to 9.9–10.2 at an AA dosage of 19–20%. Compared to kraft cooking (●) with the same AA dosage, the kappa number was lower by approximately nine points, but high yields of 40.5–40.9% were obtained, which was approximately four points higher than the soda-SAQ cooking, namely 36.5–37.2% (*p* < 0.05). Utami et al. [29] also reported high kappa numbers for the prehydrolysis kraft compared to the prehydrolysis soda-SAQ cooking of *A. crassicarpa* at an AA dosage of 17–20%. 

The kappa number obtained from soda cooking with 0.1% SAQ was approximately 30% lower than soda cooking (▲) at the same AA dosage, namely 8.6–10.2 and 19–25%, respectively (*p* < 0.05). Furthermore, the yield recorded was approximately 6% higher than soda cooking at AA dosage of 19–20%, namely 26.5–36.5% and 33.9–35.3%, respectively. This finding indicates that the soda-SAQ was more effective at *p* < 0.05. Jahan and Mun [56] stated that the soda-anthraquinone (AQ) process is suitable for non-wood cooking due to relatively low-average molecular weight of lignin. Several studies revealed that soda-AQ provided better results than soda cooking for pulp production. For example, Salehi et al. [26] reported that the AQ use in the process for wheat and rye straw increased the screen pulp yields by 2–14% and simultaneously enhanced the delignification rate by approximately 30% compared to soda cooking without AQ. Similar results were also obtained in other studies where there was an increase in pulp yield and a decrease in kappa number for cotton stalks at 0.075–0.2% AQ dosage [27] as well as bast kenaf fibers at 0.2% AQ dosage [28]. The higher pulp yield in soda-AQ cooking was caused by the retention of hemicellulose and cellulose along with the enhancement of lignin degradation. Although Utami et al. [29] reported comparable yields of *A. crassicarpa* soda-SAQ pulp to soda pulp, lower kappa numbers were observed after adding 0.1% SAQ. Additional decreases after the prehydrolysis soda cooking with AQ were also observed by Jahan et al. [57] for *A. auriculiformis* soda-AQ pulp.

The results obtained for NPF were comparable to those of Harsono et al. [34] for oil palm empty fruit bunches with a kappa number of 9.4–10.7 and 29.0–33.5% pulp yield using prehydrolysis soda-SAQ cooking, namely 3 h prehydrolysis at 150 °C and 3 hours of cooking at 160 °C with a 19–21% AA dosage. Putra et al. [58] used the same conditions as Harsono et al. [34] and obtained a 31.1% yield of pulp with a kappa number of 9.6 at 20% AA dosage for *A. crassicarpa*. Furthermore, Maryana et al. [32] recorded 33.9–34.7% yields with low kappa numbers of 5.3–5.7 using prehydrolysis for 3 h at 150 °C and soda-SAQ cooking for 2 h at 150 °C with a 17–19% AA dosage for sugarcane bagasse. Chem et al. [30] reported a higher kappa number of 12.8–22.9 from Moso bamboo stem pulp than this study, but the yields of 32.6–34.0% were comparable when using 3 h prehydrolysis at 150 °C and 3 h cooking at 160 °C with 17–23% AA dosage. Banana plant stem gave high kappa numbers of 28.3–37.6 and 27–30% pulp yields using prehydrolysis for 3 h at 150 °C and cooking for 0.5–1.5 h at 150 °C with 25% AA dosage [54]. The high residual lignin content indicated by the kappa number in Moso bamboo and banana plant stem was caused by the initial contents of the two raw materials. The yields obtained from non-wood raw materials are comparable and depend on the selected/optimum condition of the process as well as the initial α-cellulose content of the materials. Meanwhile, the kappa number recorded seemed to be affected by the initial lignin content. The comparison results between prehydrolysis soda-SAQ and prehyrolysis kraft/soda of nipa palm fronds definitely provide new information to the available literature, because no previous studies were found. 

### 2.4. Application to Totally Chlorine-Free Bleaching

The combination of prehydrolysis for 3 h and soda-SAQ cooking for 1.5 h produced 26.5–36.9% pulp yields with a low kappa number of 8.6–11.0 for totally chlorine-free bleaching. NPF pulp obtained through prehydrolysis and soda-SAQ cooking at a 20% AA dosage was selected for application in TCF bleaching because the conditions produced a kappa number of 9.9 ± 0.1, a yield of 36.5 ± 0.6%, 34.8 ± 0.5% ISO brightness, and 22.1 ± 0.7 cP viscosity, as shown in Table 2.

The optimum conditions for the soda-SAQ and TCF bleaching produced kappa numbers < 10 and a reasonable level of pulp yield, as suggested by previous studies [31,34]. Furthermore, there were significant differences between the screened pulp yields, brightness, and viscosity as the AA dosage was increased from 13 to 20% in the selected prehydrolysis–cooking process, namely 38.6 ± 0.7% vs. 26.5 ± 0.4%, 29.6 ± 0.4% vs. 35.1 ± 0.6%, and 35.1 ± 0.6 cP vs. 20.6 ± 0.5 cP, respectively (*p* < 0.05).

**Figure 6:**
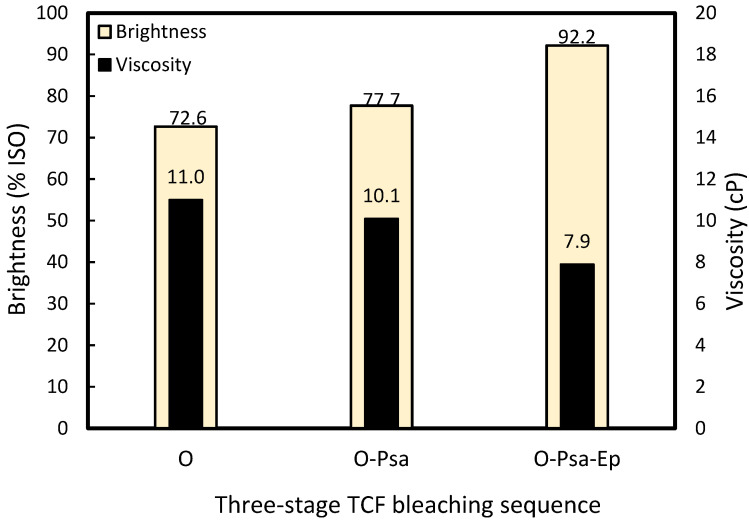
The brightness and viscosity profiles during three-stage totally chlorine-free bleaching of *Nypa fruticans* frond pulp at 20% AA dosage (O: oxygen; P_sa_: peroxymonosulfuric acid; E_p_: alkaline hydrogen peroxide).

**Figure 7:**
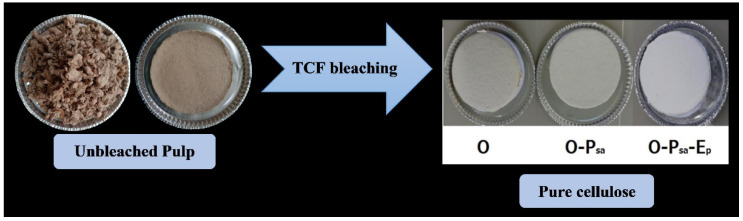
Transformation of the *Nypa fruticans* unbleached pulp into pure cellulose during three-stage totally chlorine-free bleaching at 20% AA dosage (O: oxygen; P_sa_: peroxymonosulfuric acid; E_p_: alkaline hydrogen peroxide).

In this study, the bleaching procedure proposed by previous studies [31,32,34] with a five-stage sequence of O-P_sa_-E_p_-P_sa_-E_p_ was modified to increase the dosage of NaOH in the O-stage to 2%. P_sa_ was selected instead of ozone because of its better selectivity in TCF bleaching [33]. Modifying the NaOH dosage can shorten the stages involved (O-P_sa_-E_p_) and thus reduce the number of processes, as shown in Figure 6 and Figure 7. There was also a gradual increase in pulp brightness at each stage starting from 72.6 ± 0.8 % ISO after the O stage, followed by 77.7 ± 0.7% ISO at the P_sa_ stage, and 92.2 ± 1.0 % ISO at the third E_p_ stage. Meanwhile, the viscosity decreased from 11.0 ± 0.4 to 7.9 ± 0.6 cP after the whole process. There was clear evidence of differences in the brightness and viscosity values between the two consecutive bleaching stages (*p* < 0.05). The viscosity is related to the degree of polymerization (*DP*, Equation (1)) with values of 20.6–22.3 after pulping followed by a subsequent decrease to 7.2–10.3 after TCF bleaching, which indicates the degradation of NPF cellulose fibers after these processes. The normal DP values in wood are reported to be between 300 and 1700 [59]. Similar results were obtained in the five-stage TCF sequence for sugarcane bagasse pulp, namely 89.1% ISO with 6.4 cP [23] as well as *E. globulus* pulp with 88.4% ISO and 6.0 cP [31].

The α-cellulose and ash contents of the bleached pulp as pure cellulose in this study were 94.8 ± 2.8% and 0.2 ± 0.07%, respectively, as shown in Table 3. The final yield of the purified pulp was 28.0 ± 1.3% based on the material weight. The properties of pure cellulose include 92.2 ± 1.0% ISO brightness, 94.8 ± 2.8% α-cellulose, 7.9 ± 0.6 cP viscosity, which are acceptable levels for viscose rayon and cellulose derivatives. Regarding the ash content obtained in this study, namely 0.2 ± 0.07%, it was reported that dissolving pulps from non-woody materials with an ash content of approximately 0.7% still showed potential for the production of viscose rayon, carboxymethylcellulose, and other derivatives [60,61]. Therefore, pure cellulose was successfully produced from nipa palm fronds using prehydrolysis, soda-SAQ and TCF bleaching. The processes can further be utilized for the production of other useful products or materials. 

The results show that only a small amount of pentosan, namely 1.0 ± 0.4%, remained in the purified pulp. Most of the pentosan in the materials can be isolated from the prehydrolysate for furfural production. From the sulfur-free soda cooking liquor, dissolved lignin can easily be precipitated based on a proposed method, such as the lignoboost process for raw materials of biopolymers [62]. The sulfur-free and totally chlorine-free processes for cellulose purification from NPF can be developed in future into a promising biorefinery.

## 3. Materials and Methods

### 3.1. Materials

**Figure 8:**
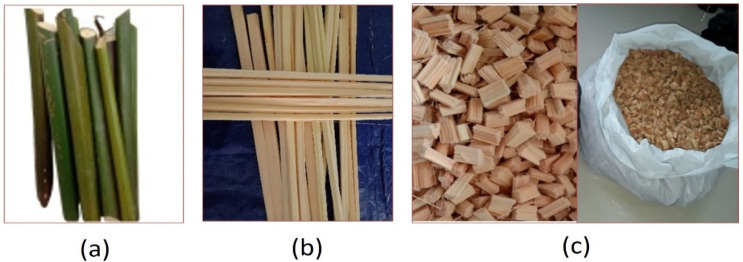
Nipa palm fronds received (**a**); peeled (**b**), and; chipped (**c**).

*Nypa fruticans* fronds were collected from Muntai village, Bantan district of Bengkalis island, Indonesia. The fiber fragments with lengths of 0.5–1.0 cm were prepared after washing and sun-drying to approximately 90%. Figure 8a–c shows the nipa palm fronds and their chipped form. SAQ (1,4-dihydro-9,10-dihydroxyanthraxene sodium salt) was provided by Air Water Performance Chemical Inc., Kawasaki, Japan. Furthermore, P_sa_ (H_2_SO_5_) was synthesized based on the method proposed by Kuwabara et al. [63] using 98% sulfuric acid (Wako Pure Chemical Industries, Ltd., Osaka, Japan) and a 50% hydrogen peroxide aqueous solution (Mitsubishi Gas Chemical Company, Inc., Tokyo, Japan) at a molar ratio of 1:3 and 70 °C.

### 3.2. Methods

#### 3.2.1. Prehydrolysis and Sulfur-Free Cooking

The prehydrolysis and cooking of the NPF were carried out in a 350 mL stainless-steel reactor (Taiatsu Techno Corporation, Tokyo, Japan), with a maximum temperature of 150 °C. The parameters used for the process include a distilled water to biomass ratio of 7 L/kg, a temperature of 150 °C, and a reaction time of 1–3 h. The wet solid residues without washing were subjected to soda-SAQ cooking using sodium hydroxide solution as fresh alkaline cooking liquor. The parameters used were a 0.1% SAQ dosage on the raw material weight, a 7 L/kg liquor to solid ratio, a temperature of 160 °C, 1–1.5 h cooking time, and active alkali (AA), namely Na_2_O, with dosages of 13, 17, 19, 20, and 25%. The residue obtained was processed into pulp using a disintegrator (FRANK-PTI GmbH), followed by screening, washing, and drying at 105 °C. Subsequently, the yield (%) of pulp was determined based on the raw materials’ weight. The optimal prehydrolysis and cooking times were selected based on the yield and kappa number. The selected parameters, procedures, and analyses were carried out based on the method from previous studies for the production of dissolving pulp from non-wood materials with similar characteristics, such as oil palm empty fruit bunch [34]. Cooking with soda without SAQ as well as the kraft method using a mixture of sodium hydroxide and sodium sulfide (sulfidity of 30% as Na_2_O) were also carried out to compare the results from the selected soda-SAQ process.

#### 3.2.2. Totally Chlorine-Free Bleaching

A modified and simple three-stage sequence of TCF bleaching, including oxygen (O), P_sa_, and alkaline hydrogen peroxide (E_p_) treatments and procedures, was carried out for the soda-SAQ pulp based on the multi-stage sequence proposed by a previous study [34]. This TCF sequence has been suggested as a solution for the pulp and paper industry to prevent pollution during bleaching [32,34]. The conditions of each stage are presented in Table 4. Depending on the TCF bleaching stage, a pulp consistency (PC) of 10 or 30% was prepared by carefully mixing the pulps with distilled water. The particles of wet pulp were mixed with NaOH in a polyethylene bag by hand, providing limited homogeneity of the treatment specifically to the samples with 30% PC; however, successful results for bleaching were obtained in this study. The PC value was then determined with the TAPPI method T 240-om 93 [64]. Regarding the oxygen delignification step, a portion of oxygen was added to the reaction mixture at the beginning of the process until the pressure was reached, followed by the reaction at the determined temperature and time (T-t). We did not use a high-share mixer for medium-consistency oxygen bleaching. Meanwhile, at the P_sa_ stage, a target amount of P_sa_ and a small amount of NaOH aqueous solution for pH adjustment were added to the pulp suspension in a polyethylene bag at the determined T-t. With respect to the E_p_ stage, target amounts of H_2_O_2_ and NaOH were also added to the pulp suspension in a polyethylene bag and at the determined T-t. These two later steps of bleaching at lower consistency (10%) were carried out for easier comparison with past results in the literature (Appendix A). The brightness and viscosity after each stage were obtained using methods explained in the next section. 

#### 3.2.3. Chemical Analysis of Materials and Pulp

For raw materials, the contents of holocellulose, alpha (α)-cellulose, pentosan, acid-insoluble lignin (Klason lignin), extractives, and ash were determined using the TAPPI methods, namely T 249 om-00, T 203 cm-99, T 223 cm-10, T 222 om-06, T 204 cm-07, and T 211 om-07, respectively [65,66,67,68,69,70]. Meanwhile, the acid-soluble lignin was determined with UV-vis spectrophotometry at 205 nm using a gram extinction coefficient of 110 L/g cm [TAPPI method: T 250 um-62]. The methods T236 om-99, T452 om-08, and T230 om-04 were used to determine the kappa number, brightness, and viscosity, respectively [71,72,73,74]. The pulp viscosity was also converted into the degree of polymerization or *DP* based on the equation proposed by Shi et al. [75] (Equation (1)):

(1) $$DP^{0.905}=0.75V$$

where *V* represents the pulp viscosity (mPa·s or cP) after the process.

#### 3.2.4. Statistical Analysis

In this study, at least two independent experiments and two measurements for each were carried out for each condition/method. The average values ± standard deviations were calculated and presented. Furthermore, a *t*-test with significance at *p* < 0.05 was used to compare any two pulp yield or kappa number values with different processing conditions/methods (Systat v13.2.01, Systat Statsoft Inc., San Jose, CA, USA), as described by Hart and Sharp [76]. It was also used to compare any two brightness or viscosity values in the TCF bleaching experiment.

## 4. Conclusions

*Nypa fruticans* frond waste is a promising raw material for producing pure cellulose and its derivatives, as well as biofuels and chemicals, due to a reasonable amount of α-cellulose and a relatively low lignin content, namely 37.3% and 18.3%, respectively. The selected prehydrolysis sulfur-free soda process with soluble anthraquinone (SAQ) catalyst has more advantages compared to the kraft and soda processes due to the decrease in the kappa number by ≥3.6 points at a 19–20% active alkali or AA dosage. Soda-SAQ also exhibited higher pulp yields of 6% at the same dosage. The optimal conditions for the soda-SAQ process include 3 hours of prehydrolysis at 150 °C and cooking for 1.5 h at 160 °C with a 20% AA concentration. Prehydrolysis sulfur-free soda cooking with the SAQ catalyst followed by three-stage totally chlorine-free bleaching with oxygen, peroxymonosulfuric acid, and alkaline hydrogen peroxide stages can produce pure cellulose as a dissolving pulp from NPF waste. The final properties of the product obtained include 92.2% ISO brightness, 94.8% α-cellulose, 7.9 cP viscosity, and 0.2% ash content. The results show the benefits and details of the prehydrolysis sulfur-free soda process with SAQ to produce pure cellulose that is acceptable for viscose rayon and cellulose derivatives. Further studies can be conducted to investigate and develop its potential for biorefinery because 99% of the pentosan was removed in the prehyrolysate and cooking liquor. The production of the viscose rayon and cellulose derivatives, such as cellulose nitrate/acetate from the obtained prehydrolysis soda-SAQ pulps, can also be explored. 

## Figures and Tables

**Table 1:** Chemical composition of *Nypa fruticans* fronds used in this study and the literature.

Components (%)	This Study *	Tamunaidu and Saka (2011) [17]
Holocellulose	61.3 ± 2.4	61.5
α-Cellulose	37.3 ± 2.1	35.1
Pentosan	24.0 ± 1.9	26.4
Acid-insoluble lignin	17.5 ± 0.7	17.8
Acid-soluble lignin	0.8 ± 0.5	1.9
Ash	16.5 ± 2.2	11.4–11.7
Extractives	1.5 ± 0.6 (Dichloromethane)	1.9 (Acetone)

* Values presented for this study are averages ± standard deviations.

**Table 2:** The brightness and viscosity of *Nypa fruticans* frond pulp obtained through prehydrolysis for 3 h at 150 °C followed by soda-SAQ cooking for 1.5 h at 160 °C *.

Active Alkali Dosage (%)	Screened Pulp Yield (%)	Brightness (% ISO)	Viscosity (cP)
13	38.6 ± 0.7	29.6 ± 0.4	21.8 ± 0.4
17	37.5 ± 0.3	32.1 ± 0.6	22.7 ± 0.4
19	37.2 ± 0.4	34.2 ± 0.4	23.0 ± 0.3
20	36.5 ± 0.6	34.8 ± 0.5	22.1 ± 0.7
25	26.5 ± 0.4	35.1 ± 0.6	20.6 ± 0.5

* Values presented are average ± standard deviations.

**Table 3:** Chemical composition of pure cellulose from *Nypa fruticans* fronds.

Components	α-Cellulose (%)	Pentosan (%)	Ash (%)
Materials	37.3 ± 2.1	24.0 ± 1.9	16.5 ± 2.2
Sulfur-free unbleached pulp	84.1 ± 2.7	3.3 ± 1.2	1.0 ± 0.5
Totally chlorine-free pulp	94.8 ± 2.8	1.0 ± 0.4	0.2 ± 0.07

**Table 4:** Conditions of three-stage totally chlorine-free bleaching for *Nypa fruticans* frond soda pulp.

Bleaching Stage	Conditions
O: Oxygen	O_2_ pressure: 0.5 MPa, NaOH dosage: 2.0%, 60 min, 115 °C, pulp consistency (PC): 30%.
P_sa_: Peroxymonosulfuric acid	H_2_SO_5_ dosage: 0.2%, 70 min, 70 °C, pH 3.0, PC: 10%.
E_p_: Alkaline hydrogen peroxide	H_2_O_2_ dosage: 2.0%; NaOH dosage: 1.4%, 60 min, 70 °C, PC: 10%.

**Figure A1:**
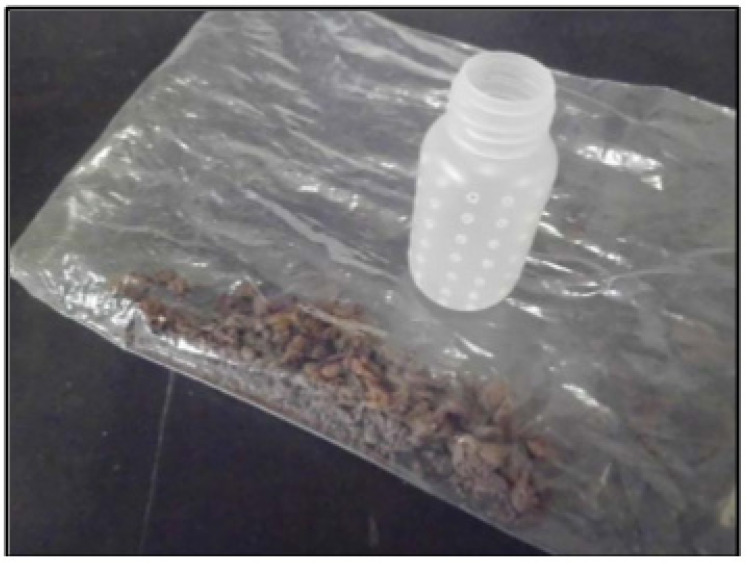
Put pulp in polyethylene plastic bag and make pulp into small particles (diameter 5–10 mm).

**Figure A2:**
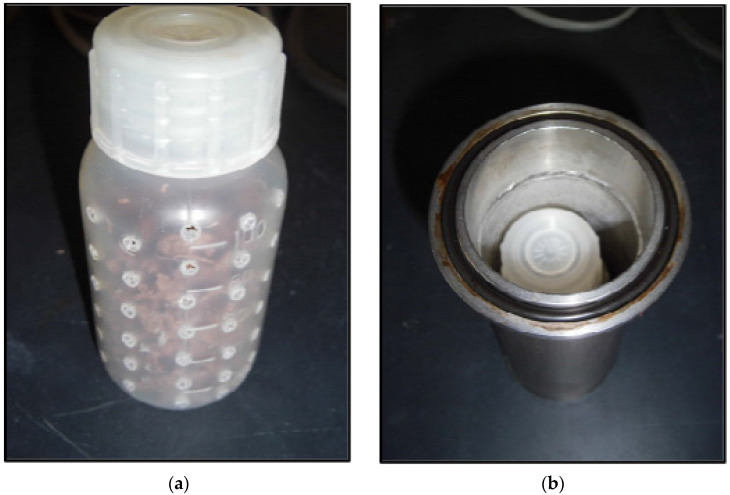
(**a**) Put pulp in a polypropylene plastic bottle, and (**b**) then put the plastic bottle into the reactor.

**Figure A3:**
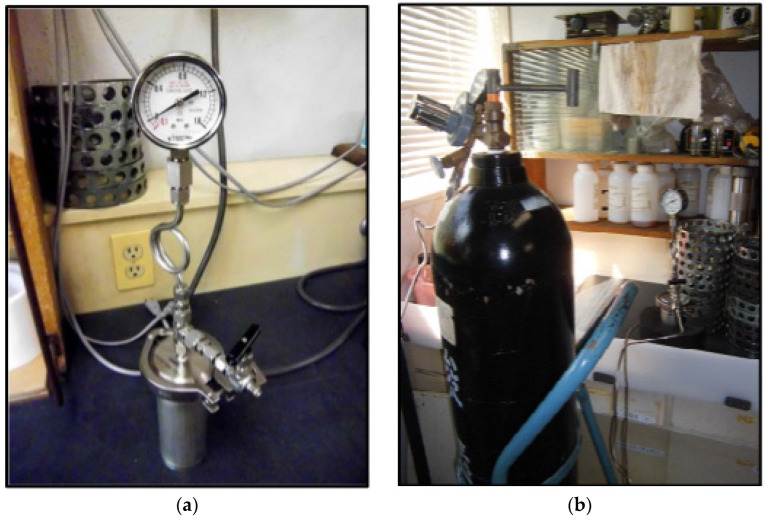
Charge oxygen gas under 0.35–0.50 MPa at room temperature.

## Data Availability

Not applicable.

## Appendix A

**High Consistency of Oxygen Bleaching**

1. Measure the water content of the air-dried pulp sample.
2. Measure the pulp sample with 10.0 g of oven-dried weight, and then tear it, and put them in a polyethylene plastic bag.
3. Prepare 20 mL (or A: 10 mL for MgSO4·7H2O2 and B: 10 mL for NaOH) of distilled water for 30% of pulp consistency.
4. Prepare the targeted NaOH and Mg dosage. Example 1.0% and 0.1% respectively.
5. If the pulp sample was 10.0 g, then add 2.5 mL of 1 N (1 mol/L) NaOH (0.1 g of NaOH) into the distilled water.
6. If the NaOH is 2N (2 mol/L), then add 1.25 ml.
7. Drop A: 10 mL of Mg solution containing 100 mg of MgSO4·7H2O2 to the pulp sample, and mix the pulp sample in a polyethylene bag with hands (Figure A1). Drop B: 12.5 mL or 11.25 mL of the NaOH solution to the pulp sample, and mix the pulp sample in a polyethylene bag with hands (Figure A1).
8. Make pulp into small, bulky and fluffy particles (diameter 5–10 mm).
9. Put pulp in a polypropylene plastic bottle and put it in a reactor (Figure A2).
10. Charge oxygen gas into the reactor under 0.35–0.50 MPa (3.5–5.0 bars) at room temperature for several minutes (Figure A3). Release the oxygen gas from the reactor, and repeat the charging twice for purging air inside sufficiently.
11. Set the reactor in 123 °C oil bath.
12. Count the reaction time from 91–115°C.
13. Cool the reactor after 60 minutes in a water bath.
14. Transfer the pulp into a jar of a disintegrator and wash the pulp with 1.5 L of water. Dewater the pulp suspension using a Büchner funnel.
15. Wash the pulp twice more, and make 2 pieces of hand-sheets and air-dry them.
